# Anti-PD-1 antibody armored γδ T cells enhance anti-tumor efficacy in ovarian cancer

**DOI:** 10.1038/s41392-023-01646-7

**Published:** 2023-10-20

**Authors:** Yue Wang, Jingyi Han, Dongdong Wang, Menghua Cai, Yi Xu, Yu Hu, Hui Chen, Wei He, Jianmin Zhang

**Affiliations:** 1 Department of Immunology, CAMS Key Laboratory of T-cell and Immunotherapy, Institute of Basic Medical Sciences, Chinese Academy of Medical Sciences and School of Basic Medicine, Peking Union Medical College, State Key Laboratory of Common Mechanism Research for Major Diseases, Beijing, 100005 China; 2 Department of Thoracic Surgery, Qilu Hospital of Shandong University, Jinan, Shandong 250012 China; 3 Haihe Laboratory of Cell Ecosystem, Chinese Academy of Medical Sciences & Peking Union Medical College, 288 Nanjing Road, Tianjin, 300020 China; 4 Changzhou Xitaihu Institute for Frontier Technology of Cell Therapy, Changzhou, 213000 China

**Keywords:** Immunotherapy, Tumour immunology

## Abstract

γδ T cells have the unique ability to detect a wide range of tumors with low mutation burdens, making them attractive candidates for CAR-T-cell therapy. Unlike αβ T cells and other immune cells, γδ T cells are superior in MHC non-restriction, selective cell recruitment, and rapid activation. However, clinical trials have shown limited clinical benefits, and the adoptive transplantation of γδ T cells has often fallen short of expectations. We hypothesized that the limited effectiveness of γδ T cells in eradicating tumor cells may be attributed to the inhibitory tumor microenvironment induced by the suppressive PD-1/PD-L1 axis. Herein, we constructed novel armored γδ T cells capable of secreting humanized anti-PD-1 antibodies, referred to as “Lv-PD1-γδ T cells. Lv-PD1-γδ T cells showed improved proliferation and enhanced cytotoxicity against tumor cells, resulting in augmented therapeutic effects and survival benefits in ovarian tumor-bearing mice. These engineered cells demonstrated a prolonged in vivo survival of more than 29 days, without any potential for tumorigenicity in immunodeficient NOD/SCID/γ null mice. We also found that Lv-PD1-γδ T cells exhibited excellent tolerance and safety in humanized NOD/SCID/γ null mice. With attenuated or eliminated immunosuppression and maximized cytotoxicity efficacy by the local secretion of anti-PD1 antibodies in tumors, Lv-PD1-γδ T cells can serve as a promising “off-the-shelf” cell therapy against cancers.

## Introduction

Investigations of the functional plasticity and phenotypic heterogeneity of human γδ T cells are currently flourishing.^1^ Unlike αβ T cells, their features include major histocompatibility complex (MHC)-independent characteristics and an abundance of antigen recognition.^2^ Thus, γδ T cells are considered as attractive “off the shelf” candidate for allogenic cell therapy. As an important type of unconventional effector cell, γδ T cells can be rapidly recruited into the tumor microenvironment (TME), acting as cytotoxic cells to mediate tumor immune surveillance.^3,4^ Clinical trials conducted over the past decade have demonstrated that γδ T cell-based immunotherapies are safe and well tolerated.^5^ The most common adverse events include systemic fatigue, fever and chills, and flu-like symptoms. There have been few recorded grade 3/4 or life-threatening adverse events.^6^ The pharmacodynamic activation of γδ T-cell allogeneic transplantation or in vivo expansion has been firmly established in patients with myeloma, lymphoma, liver cancer, ovarian cancer, and lung cancer.^7–11^ Most patients progressed to at least a partial response on γδ T adoptive cellular immunotherapy. Some patients with advanced lung cancer achieved durable remission and improved overall survival.^8^ However, the therapeutic efficacy of γδ T cells in the clinic is variable.^12^ γδ T cells show high flexibility and are easily polarized into regulatory T-cell phenotypes when exposed to a complicated TME. To address this issue, strategies involving combination therapies or genetic manipulation of γδ T cells are imperative to improve the required intrinsic antitumor function.

Programmed death receptor 1 (PD-1, CD279) is a crucial immunosuppressive receptor on the surface of kinds of immune cells and myeloid cells.^13^ PD-1 was initially discovered to be expressed on activated or differentiated immune cells, which restricts cell over-activity and prevents tissue damage.^14^ In contrast, the expression of PD-1 ligands (CD274/PD-L1 and CD273/PD-L2) is one of the defensive mechanisms by which tumor cells protect themselves from tumor-reactive T cells.^15^ PD-1 interacts with its ligands to deliver inhibitory signals through the phosphorylation of PD-1 peptide within the immunoreceptor tyrosine-switch motif (ITSM). By recruiting SHP phosphatase activity (PTPN11/SHP-2), PD-1 directly suppresses T-cell receptor (TCR) signaling. PD-1 signal transduction inhibits phosphorylation of the CD247/CD3ζ immunoreceptor tyrosine-based activation motif (ITAM) sites and weakens the activation of ZAP70 and PKCθ.^16^ Within the TME, the PD-1/PD-L1 axis represents a major inhibitory pathway for adaptive immunotherapy against tumors, which attenuates the TCR-mediated production of interleukin-2 (IL-2) as well as the proliferation of the T cells.^17^ Studies in non-small cell lung cancer (NSCLC) have shown that the PD-1/PD-L1 axis induces effector αβ T-cell disability or regulatory T cells (Tregs) generation, thereby limiting their infiltration in tumor tissues.^18^ Active PD-1 signaling maintains the inhibitory activity and survival of Forkhead box protein P3 (FOXP3^+^) Tregs.^19^ Iwasaki et al. reported that PD-1/PD-L1 interaction network transmitted coinhibitory signals in γδ T cells, where IFN-γ production and cytotoxicity were significantly reduced.^20^ In some patients with follicular lymphoma, the high PD-1^+^ ratio on the surface of infiltrating γδ T lymphocytes (γδTILs) was related to malignant tumor immune escape.^21^ In response to tumor cells, γδ T cells upregulate the levels of PD-1 expression, leading to apoptosis and even collapse, which is a major obstacle to the complete activation and cytotoxicity of γδ T cells. This likely explains that the average response rate and average clinical benefit rate of γδ T-cell immunotherapy have failed to meet expectations in many clinical trials.^22^ In addition, inflammatory γδ T cells can indirectly inhibit αβ T-cell activation through the PD-1/PD-L1 axis, leading to a decrease in tumor-infiltrating lymphocytes.^23^ All the above checkpoint toxicity exacerbates the plasticity of effector T cells.

Currently, as a broad-spectrum antitumor drug for balancing immune checkpoints, PD-1 antibody has displayed notable therapeutic efficacy in patients with NSCLC, melanoma, and bladder cancer.^24,25^ However, not all patients respond to a single therapy. To enhance and broaden the efficiency of immune checkpoint blockade with monoclonal PD-1 antibodies, various combinational immunotherapies have shown better clinical benefits. An example of this is the success of the combination of Vγ9Vδ2 T cells and therapeutic PD-1 monoclonal antibodies (mAbs) that enhanced the cytotoxicity of Vγ9Vδ2 T cells.^26–28^ In another preclinical experiment of PC-3 prostate tumors treated with Vγ9Vδ2 T cells, anti-PD-1 mAbs also enhanced the immune activity effectiveness of Vγ9Vδ2 T cells and reduced the tumor volume of tumor-bearing mice to almost zero after 5 weeks.^29^

In this study, we prepared genetically engineered γδ T cells secreting PD-1 antibodies to treat ovarian cancer. Armored γδ T cells exhibited enhanced cytotoxicity in vitro and strong inhibition in transplanted ovarian tumor models in immunodeficient mice. This novel alternative approach provides new ideas and a theoretical basis for the development of combined immunotherapy for tumors.

## Results

### PD-1 expression is upregulated during γδ T-cell activation

To examine the dynamics of PD-1 expression on γδ T cells during in vitro expansion, freshly isolated human peripheral blood mononuclear cells (PBMCs) were used to expand γδ T cells in anti-TCR pan-γδ antibody-coated plates. Flow cytometry results showed that PD-1 expression was rapidly induced within 48~72 h after activation by the anti-TCR pan-γδ antibody (Supplementary Fig. 1a–c). After the cells were transferred to the wells without coating with anti-TCR pan-γδ antibody, PD-1 expression gradually decreased and stabilized to the initial level (Supplementary Fig. 1b, c). Moreover, within 12 h of exposure to HepG2 liver cancer cell line, a significant increase was observed in the level of PD-1 expression on γδ T cells (Supplementary Fig. 1d, e). Taken together, these results suggest that γδ T cells are regulated by the PD-1 signaling pathway when they are activated or they are killing tumor cells, indicating the potential benefit of combination therapy with γδ T cells and PD-1 immune checkpoint inhibitors.

### Preparation of genetically engineered γδ T cells secreting anti-PD-1 antibodies

**Fig. 1:**
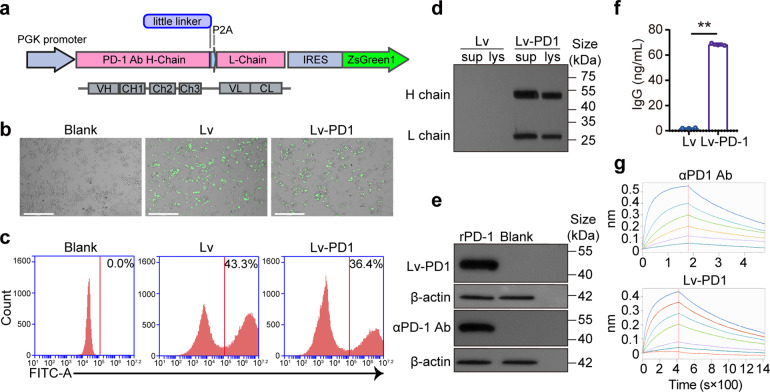
Preparation of Lv-PD1-γδ T cells secreting specific anti-PD-1 antibodies. **a** Schematic representation of the lentiviral vector encoding anti-human PD-1 antibody. The signal peptide was linked to the full-length variable heavy sequence, serine glycine little linker, P2A element, and variable light chain sequence. The P2A peptides allowed control of the HC and LC ratio to achieve better “self-cleavage” efficiency and higher antibody expression levels. **b** Representative images showing fluorescence detection in Lv-γδ T cells or Lv-PD1-γδ T cells over 72 h. Scale bars, 275 μm. **c** Representative flow cytometry histograms depicting the infection efficiency of γδ T-cell transduction, as detected by fluorescent ZsGreen^+^ cells. **d** Western blot of supernatants (sup) and whole-cell lysates (lys) from equivalent numbers of transduced γδ T cells stained with an anti-IgG antibody. The data demonstrate a ~55-kDa heavy chain and a ~25-kDa light chain. **e** The αPD-1 antibody was used as a positive control, and Lv-PD1-γδ T cells were cocultured with HEK-293T cells overexpressing PD-1 protein (rPD-1) or HEK-293T cells (Blank). Western blotting was performed to investigate the specificity of the expressed PD-1 antibody. β-actin was used as a protein loading control. **f** ELISA to measure the levels of the secreted PD-1 antibody in cell culture supernatants from transduced γδ T cells (*n* = 3). Data are represented as the mean ± SEM. **p < 0.01. **g** ForteBio Octet analysis was designed to illustrate the binding affinity of the supernatant antibody to the serially diluted purified PD-1 protein

To prepare genetically engineered γδ T cells secreting anti-PD-1 antibodies, a full-length humanized PD-1 antibody sequence was constructed in a lentiviral plasmid with ZsGreen (Fig. 1a). The sequences for PD-1 antibody heavy chain (HC) and light chain (LC) were separated by porcine teschovirus-1 (P2A) peptide for the generation of heavy chain and light chain separately. The lentivirus was packaged in HEK-293T cells, and the virus titer was 8E + 08 (Supplementary Fig. 2). Then, we applied the lentivirus to infect γδ T cells. Fluorescence microscopy and flow cytometry showed that the infection efficiency of the ZsGreen^+^ Lv-PD1-γδ T cells was approximately 40% (MOI = 12) (Fig. 1b, c). The production of PD-1 antibodies by Lv-PD1-γδ T cells was verified by Western blotting (Fig. 1d). The antibody exhibited the ability to recognize the PD-1 proteins (Fig. 1e). The concentration of human IgG in the supernatant was approximately 77 ng/mL after 72 h of culture (Fig. 1f). The ForteBio results showed a slightly lower binding affinity [KD (*M*) = 5.50E*-*09] for Lv-PD1-γδ T cells compared with the commercial anti-PD-1 antibody (αPD-1) [KD (M) = 1.64E*-*10] (Fig. 1g). In summary, prepared Lv-PD1-γδ T cells secreted functional anti-PD-1 antibodies.

### Lv-PD1-γδ T cells exhibit improved activation, increased production of cytokines, and enhanced cytotoxicity

**Fig. 2:**
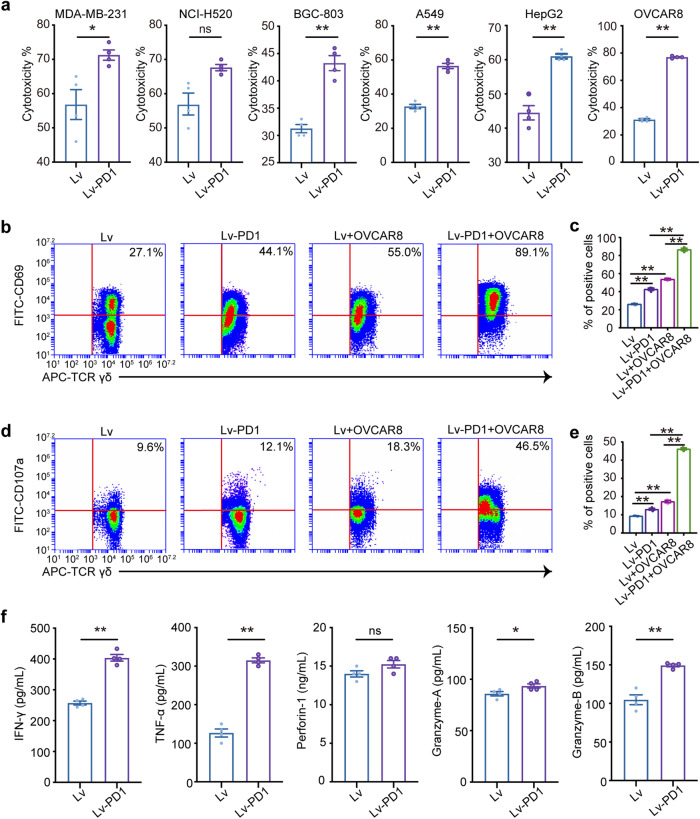
Lv-PD1-γδ T cells exhibit enhanced cytotoxicity to tumor cells. **a** Cytotoxicity assay demonstrating increased lysis of tumor cells by Lv-PD1-γδ T cells (*n* = 3). **b**–**e** Representative plot and quantification of flow cytometric analysis demonstrating the expression levels of CD69 (**b**, **c**) and CD107a (**d**, **e**) on γδ T cells cocultured with OVCAR8 tumor cells for 4 h at an effector: target ratio (E: T) of 10: 1 (*n* = 3). **f** Supernatants from cocultures of γδ T cells with OVCAR8 cells for 4 h were analyzed in vitro for cytokines (*n* = 3). ELISA to measure the expression levels of IFN-γ: **p < 0.01; TNF-α: **p < 0.01; perforin-1: ns, no significant difference; granzyme A: *p < 0.05; granzyme B: **p < 0.01. Data are represented as the mean ± SEM by a two-tailed unpaired *t* test

Next, we examined the cytotoxicity of Lv-PD1-γδ T cells to different tumor cells by measuring the lactate dehydrogenase (LDH) level (Fig. 2a). The results showed that compared with natural γδ T cells, Lv-PD1-γδ T cells were superior in terms of antitumor ability in vitro, including in liver cancer (HepG2), ovarian cancer (OVCAR8), gastric cancer (BGC-803), lung cancer (A549), and breast cancer cells (MDA-MB-231). We also found that in the early stage of killing activity, the levels of the active inducer molecule CD69 and degranulation-related molecule CD107a were significantly higher on Lv-PD1-γδ T cells than those on Lv-γδ T cells (Fig. 2b–e). Meanwhile, enzyme-linked immunosorbent assay (ELISA) results showed that after coincubation with OVCAR8 cells for four hours, Lv-PD1-γδ T cells released more antitumor cytokines including TNF-α, IFN-γ, and granzyme A/B (Fig. 2f). The above results demonstrated that Lv-PD1-γδ T cells yielded incremental improvements in activation and cytotoxicity.

### Lv-PD1-γδ T cells have no potential tumorigenicity

**Fig. 3:**
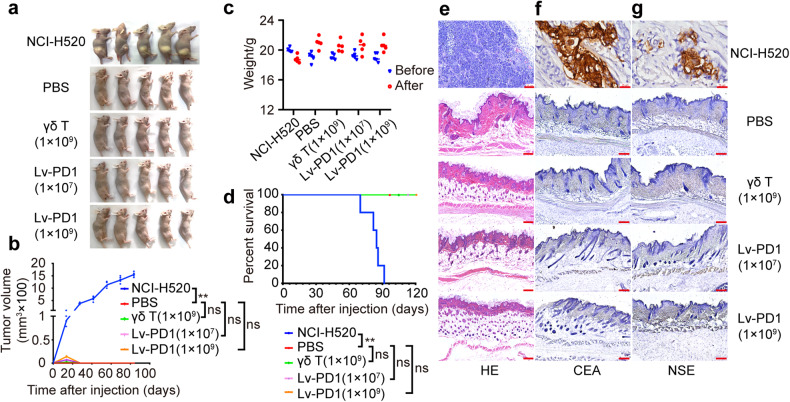
Lv-PD1-γδ T cells have no potential tumorigenicity. **a** Imaging of the growth status of nude mice subcutaneously injected with NCI-H520 (8 × 10^6^ cells/mouse), 1× PBS (50 μL), high-dose γδ T cells (1 × 10^9^ cells/mouse), medium-dose Lv-PD1-γδ T cells (1 × 10^7^ cells/mouse), or high-dose Lv-PD1-γδ T cells (1 × 10^9^ cells/mouse) in the 12th week (*n* = 5). **b** Curves of tumor growth and quantitative statistical analysis of tumor volume over 12 weeks. Data are represented as the mean ± SEM. **p < 0.01, by multigroup *t test* statistical analysis. **c** We recorded the body weight of nude mice before and after transplantation at the final time point. **d** Survival curve and statistical analysis within 120 days, using a log-rank (Mantel‒Cox) test with a 95% CI. **e** Hematoxylin-eosin staining of the subcutaneous injection site in nude mice. Scale bars, 25 μm. **f**, **g** Immunohistochemistry for CEA (**f**) and NSE (**g**) in nude mice after subcutaneous injection. Scale bars, 25 μm

Based on the data showing the efficacy of Lv-PD1-γδ T cells, they are expected to provide potential for tumor immunotherapy. Therefore, we sought to evaluate their preclinical safety in immunodeficient mice. As shown in Fig. 3a, b, NCI-H520 cells, as a positive control, grew to tumor nodules on the epidermis at the injection sites 12 weeks after injection. No nodules were found in the high-dose γδ T group, the medium- and high-dose Lv-PD1-γδ T groups, or the negative control group. These four groups showed normal skin histomorphology. The body weight of NCI-H520 mice decreased, while those of other groups showed a slight increase (Fig. 3c). The survival curve supported the safety and showed that Lv-PD1-γδ T cells did not cause mouse death (Fig. 3d). HE staining was applied to examine whether there were tumor cells growing at the injection sites. NCI-H520 cells grew into typical tumors while no signs of tumor cells were observed in the γδ T-cell group or Lv-PD1-γδ T-cell groups (Fig. 3e). In addition, we also performed immunohistochemical staining to examine the expression of carcinoembryonic antigen (CEA) and neuron-specific enolase (NSE) for the confirmation of tumors (Fig. 3f, g). These results suggest that Lv-PD1-γδ T cells have no potential tumorigenicity.

### Distribution and migration dynamics of adoptively transferred Lv-PD1-γδ T cells

**Fig. 4:**
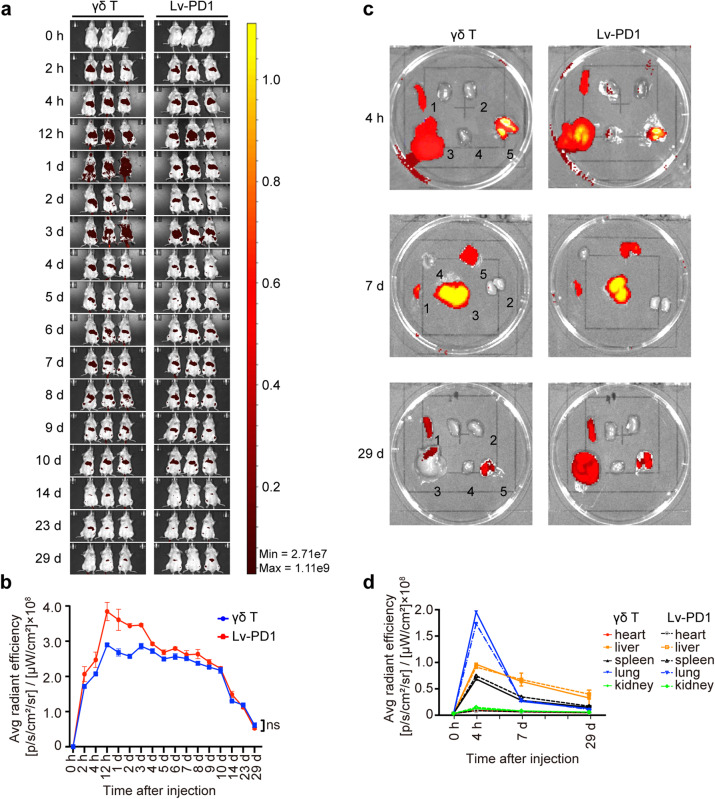
Characterization of the distribution of adoptively transferred Lv-PD1-γδ T cells in vivo. **a** Near-infrared fluorescence imaging of DiR-labeled cells in NSG mice with an IVIS Imager after i.v. injection of a single dose (1 × 10^7^) of γδ T cells or Lv-PD1-γδ T cells, shown as ventral images. **b** Quantification of the average radiant efficiency at the ventral site, as shown in **a**, within 29 days (*n* = 3). Data are represented as the mean ± SEM. ns, no significant difference. **c**, **d** Images of the major organs of each mouse were collected periodically. Ex vivo live imaging (**c**) and quantification (**d**) of DIR^+^ γδ T cells in the (1) spleen, (2) kidney, (3) liver, (4) heart, and (5) lung in NSG mice

Next, we characterized the distribution and persistence of XenoLight DiR^+^ γδ T cells injected via the caudal vein in vivo. The results showed that in NOD/SCID/γ null (NSG) mice, both γδ T and Lv-PD1-γδ T cells could be retained in vivo for ~29 days (Fig. 4a, b). For the distribution of intravenously infused γδ T cells in mice, we isolated major organs at different time points after the injection of cells. Four hours after injection, the cells mainly accumulated in the lung through a “first-pass” effect, which may involve sequestration in the capillary bed. After 7 days, almost all γδ T cells were redistributed in the liver, which served as a “reservoir” for adoptively transferred cells, and a small amount was observed in the spleen and lung (Fig. 4c, d).

### Assessment of the safety of Lv-PD1-γδ T cells

Next, we assessed the safety of Lv-PD1-γδ T cells in humanized NSG mice. To reconstitute the human immune system, 2 × 10^7^ freshly isolated human PBMCs were transfused into NSG mice (Hu-PBMC mice). Flow cytometry analysis showed that CD45^+^ immune cells reached approximately 80% in the peripheral blood of Hu-PBMC mice 1 week after transfusion (Supplementary Fig. 3a, b). To preliminarily evaluate the safety of ex vivo-expanded Lv-PD1-γδ T cells, Hu-PBMC mice received escalating doses of Lv-PD1-γδ T cells by intravenous injection into the caudal vein. The results showed that there were no acute toxic reactions, such as ataxia, tremor, convulsion, tachycardia and death, in mice infused with 1.0 × 10^7^, 3.0 × 10^7^, and 9.0 × 10^7^ cell doses. At infusion doses of up to 9.0 × 10^7^ of Lv-PD1-γδ T cells, the weight of the mice slightly decreased after 3 days but recovered quickly (Supplementary Fig. 3c). Together, Lv-PD1-γδ T cells displayed very good tolerance and safety in humanized NSG mice.

### Lv-PD1-γδ T cells significantly enhance therapeutic efficacy in ovarian tumor-bearing mice

**Fig. 5:**
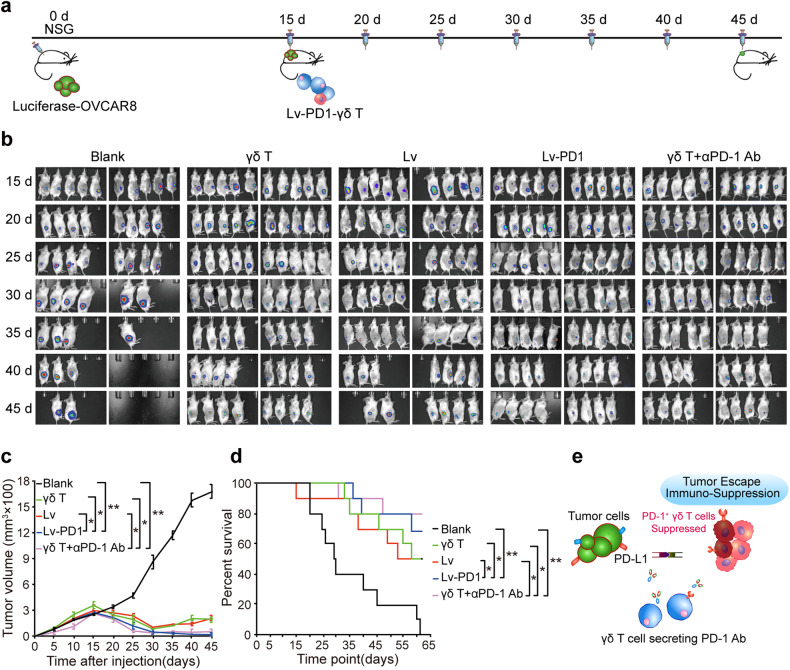
Lv-PD1-γδ T cells augmented therapeutic efficacy in ovarian tumor-bearing mice. **a** Schematic diagram of the experimental setup for constructing OVCAR8-GFP-Luc tumor-bearing mice and detecting the therapeutic efficacy of Lv-PD1-γδ T cells in vivo. **b**–**d** Imaging and quantification of γδ T-cell adoptive immunotherapy over time in xenogeneic solid tumor mice treated with 1× PBS (Blank), γδ T cells, nonsense-vector γδ T cells (Lv), PD-1-Ab-secreting γδ T cells (Lv-PD1) or γδ T cells with anti-PD-1 Ab (γδ T + αPD-1 Ab) (*n* = 10). **b** Bioluminescence imaging with an IVIS Spectrum system. **c** Curves of tumor growth and quantitative statistical analysis of tumor volume. **d** Survival curve and statistical analysis were performed using a log-rank (Mantel‒Cox) test with a 95% CI. **e** The graphical abstract of the study. Data are represented as the mean ± SEM. ns, no significant difference, *p < 0.05; **p < 0.01. Significance was calculated with Student’s *t* test and paired *t* test

Next, we sought to examine the potent functionality of Lv-PD1-γδ T cells in vivo in luciferase-OVCAR8-NSG mice in which OVCAR8-GFP-Luc cells were subcutaneously injected. Lv-PD1-γδ T cells were intratumorally injected every 5 days (Fig. 5a). As expected, γδ T cells inhibited tumor growth compared with that in the untreated group. Strikingly, the treatment effect was optimized in Lv-PD1-γδ T cells and even led to the eradication of disseminated disease in a portion of treated mice (Fig. 5b). The tumor suppression effect of Lv-PD1-γδ T cells was comparable to or even better than that in the combined group (Fig. 5b, c). The survival curve showed an improvement in the survival of Lv-PD1-γδ T cell-treated mice (Fig. 5d). Together, these findings demonstrate that Lv-PD1-γδ T cells could functionally improve the therapeutic efficacy of γδ T cells in ovarian tumor-bearing mice.

### Lv-PD1-γδ T cells are beneficial for cell infiltration, colonization, and survival in the tumor microenvironment

**Fig. 6:**
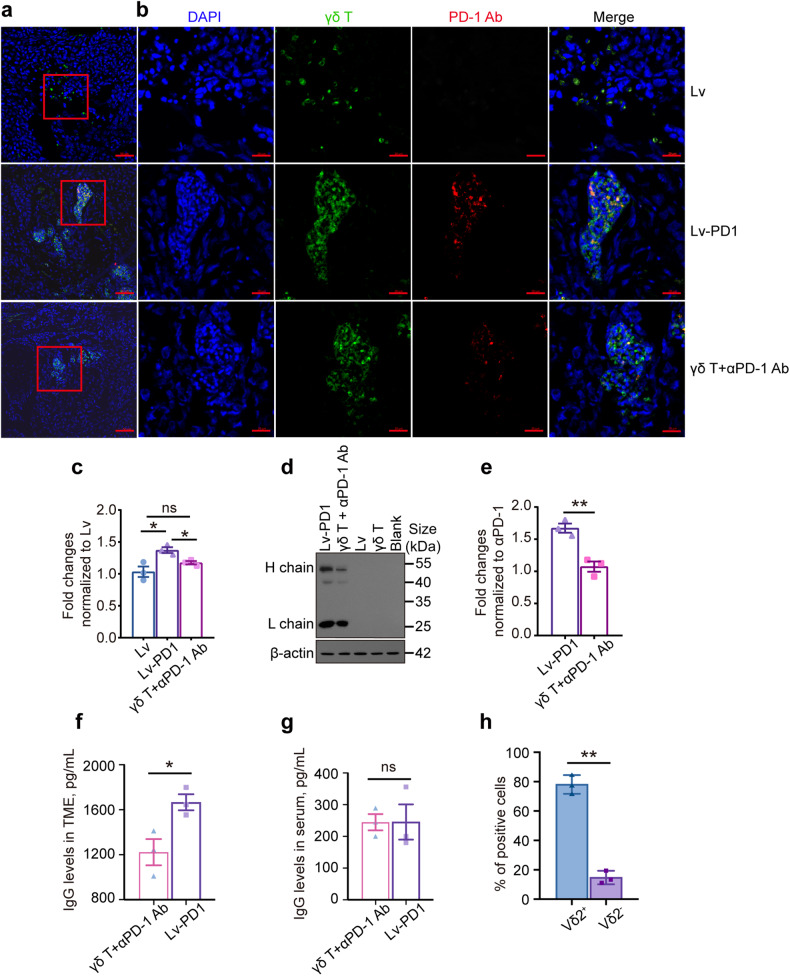
Lv-PD1-γδ T cells secreted high levels of anti-PD1 antibodies in the tumor microenvironment. **a** Immunofluorescence staining of the infiltration of γδ T cells (green) and colocalization with PD-1 Ab (red) in tumor tissues from Luciferase-OVCAR8-NSG mice treated with Lv-γδ T cells, Lv-PD1-γδ T cells and γδ T + αPD-1 Ab. Scale bars, 50 μm. **b** Observation of fluorescently labeled γδ T cells under magnification, with a scale bar of 20 μm. **c** Quantification of the number of positive cells visible per unit area as shown in **a**. Five tissue sections were counted for each group of mice. Data are represented as mean ± SEM. ns, no significant difference; *p < 0.05, by a two-tailed unpaired *t* test. **d**, **e** Western blot analysis (**d**) and quantification of IgG (**e**) to determine the abundance of PD-1 antibody in tumor tissues from luciferase-OVCAR8-NSG mice subjected to five different treatments (*n* = 3). Data are represented as the mean ± SEM. with the Lv-PD1-γδ T and γδ T + αPD-1 Ab groups. **p < 0.01, by a two-tailed unpaired *t* test. **f**, **g** ELISA for quantifying the levels of human IgG in the tumor tissues (**f**) and the serum (**g**) of treated mice. Data are represented as the mean ± SEM. ns, no significant difference; *p < 0.05. **h** Flow cytometry was used to distinguish between infiltrated Vδ2^+^ or Vδ2^−^ γδ T cells in tumor tissues. **p < 0.01, by a two-tailed unpaired *t* test

To determine how long intratumorally injected Lv-PD1-γδ T cells could survive in tumors, Western blotting was applied to detect the expression of antibody-heavy chains and light chains secreted by Lv-PD1-γδ T cells. The results showed that the secreted PD-1 antibody could be detectable in the tumor microenvironment for up to 9 days (Supplementary Fig. 4). Immunofluorescence (IF) staining was also performed to visualize the intratumorally infused γδ T cells in tumor tissues. The results showed that more aggregated Lv-PD1-γδ T cells in the TME colocalized with the PD-1 antibody, enhancing PD-1 blockade (Fig. 6a–c). Accordingly, Western blot analysis suggested that the abundance of PD-1 antibody in the Lv-PD1-γδ T group was approximately 1.53-fold higher than that in the γδ T plus PD1 antibody combined group (Fig. 6d, e). ELISA showed that the Lv-PD1-γδ T group had higher levels of human IgG in the tumor environment (Fig. 6f). There was no difference in serum levels between these two groups (Fig. 6g), indicating that Lv-PD1-γδ T cells could secrete high levels of anti-PD1 antibodies locally. Flow cytometry showed that ~78% of tumor-infiltrating γδ T cells were Vδ2^+^ γδ T cells (Fig. 6h). Taken together, these findings suggest that aggregated Lv-PD1-γδ T cells could more efficiently interrupt the PD-1/PD-L1 inhibition and dramatically boost cell infiltration and colonization. Thus, the autocrine PD-1 antibody relieved the suppressive effect of the inhibitory TME on γδ T cells and thus significantly augmented therapeutic efficacy in ovarian tumor-bearing mice.

## Discussion

It is universally known that γδ T cells applied as allogeneic immune cells have numerous distinct benefits over other effector cells. They have excellent features in terms of chemotaxis, selective cell recruitment, and conservative monomorphism characteristics.^30,31^ Activated γδ T cells are characterized by multitarget recognition, powerful killing, and no MHC restriction, which makes them favorable for allogeneic adoptive immunotherapy. Nevertheless, inhibitory PD-1/PD-L1 signaling in the tumor microenvironment strongly interferes with the efficacy of γδ T-cell adoptive immunotherapy.

Our results demonstrate that Lv-PD1-γδ T cells can generate functional anti-PD-1 antibodies to block the PD-1/PD-L1 inhibitory pathway, resulting in enhanced cytotoxicity and improved therapeutic efficacy in ovarian cancer by secreting high levels of IFN-γ, TNF-α, and granzyme A/B. As a novel cell-based type of gene therapy product, inappropriate gene integration of Lv-PD1-γδ T cells may lead to variable transformation or carcinogenesis. Therefore, we evaluated their safety and effectiveness in relevant preclinical models. Our results showed that therapy with PD-1 antibody-armored γδ T cells didn’t incur toxicity in nude mice. Lv-PD1-γδ T cells were also well tolerated in humanized NSG mice.

Activated γδ T cells in an immunosuppressive tumor microenvironment are sensitive to the PD-1/PD-L1 axis, leading to limited clinical responses. Considering the known challenges, the combination of γδ T-cell immunotherapy and PD-1 checkpoint inhibitors is a new therapeutic strategy. Our studies demonstrated that Lv-PD1-γδ T cells could functionally improve the therapeutic efficacy of γδ T cells in ovarian tumor-bearing mice even though a very subtle difference between Lv-PD1-γδ T cells and a combination of γδ T cells with a commercialized PD-1 antibody in the in vivo mouse model. We found that genetically engineered Lv-PD1-γδ T cells produced higher levels of anti-PD-1 antibodies locally than the combined group. Therefore, our strategy has the advantage that the autocrine PD-1 antibodies produced by Lv-PD1-γδ T cells could more efficiently prevent the local immunosuppression of γδ T cells. This is critical to maintain the active cytotoxicity of tumor-infiltrated γδ T cells against tumors in the clinic. One of the limitations of this study is that in vivo experiments were performed only in a mouse model of ovarian cancer. Multi-targeting studies of a wider variety of tumors will still be required in the future, which will contribute to clarifying the broad applicability of the armored Lv-PD1-γδ T cells in adoptive γδ T-cell therapy.

Human γδ T cells subsets exhibit different tissue tropisms: Vδ1-positive cells are usually enriched in mucosal tissues such as the intestine, skin, lung, vagina, and liver. γδ T cells in the blood circulation preferentially express the TCRδ2 isotype (Vδ2^+^ cells). The Vδ2^+^ cells preferred to pair with the TCRγ9 isotype (Vγ9Vδ2 T cells). Vγ9Vδ2 T cells expanded by aminobisphosphonates have been applied in many cancer clinical trials and achieved objective responses.^32^ One study by Zumwalde et al. found that compared with CD8^+^ T cells and CD4^+^ T cells, adoptively transferred Vδ2^+^ T cells were more likely to circumvent the immunosuppressive TME due to their lower PD-1 expression.^33^ It is well known that the consequences of immunotherapy are largely affected by the infiltration capacity of immune cells.^34^ In many preclinical xenogeneic solid tumor mouse models, cytotoxic Vγ9Vδ2 T cells could circulate from the bloodstream to the tumor site in both the early and late stages of cancer development.^6^ Our study demonstrated that in ovarian cancer-bearing mice, Lv-PD1-γδ T cells displayed excellent performance in tumor tissue penetration and self-colonization. The number of infiltrating Lv-PD1-γδ T cells in the TME was higher than that in the γδ T plus PD1 antibody combined group. Among them, almost all tumor-infiltrating γδ T cells belonged to the Vδ2^+^ phenotype. Therefore, it is worthwhile to imagine that the Lv-PD1-γδ T cells may elucidate better combination treatment for cancer patients with immunosuppressive microenvironments.

Promisingly, the next generation of engineered γδ T cells has evolved in recent years, including ex vivo armed γδ T cells (EATs), CAR-γδ T cells, tumor-specific Vγ9Vδ2 TCRs (TEGs), iNKT TCR transfer into γδ T cells and introduction of drug-resistance genes.^35,36^ However, γδ T cells account for only a small portion (1~5%) of the peripheral T-cell pool. Effective activation and amplification are required in vitro or in vivo for clinical benefits. Vγ9Vδ2 T cells can sense stress signals through the phosphoantigens (pAgs) on malignant cells. An innovative method is the utilization of liposome systems to encapsulate γδ T-cell sensitizers (such as zoledronate and alendronate). It is beneficial for promoting their passage through the tumor neovascularization system, enhancing infiltration as well as killing sensitivity.^37^ Interestingly, “TEG001”, a defined high-affinity Vγ9Vδ2 TCR clone 5 transfused into αβ T cells, overcame the disadvantage of weak proliferation of Vγ9Vδ2 T cells themselves and got strong anti-tumor reactivity simultaneously. It also reduced the susceptibility of Vγ9Vδ2 T cells to killer-cell Ig-like receptor (KIR) inhibition. It has been applied in phase I clinical trials in patients with myelodysplastic syndromes and refractory acute myeloid leukemia (AML).^38^ Similarly, αβ T cells engineered to secrete antibodies were used for a diverse set of therapeutic strategies.^39^ For instance, previous studies reported that targeted delivery of a PD-1-blocking single-chain antibody fragment (scFv) by CAR-T cells enhanced antitumor efficacy in vivo.^40^ These cells may optimize clinical applications and generate novel concepts for adoptive immunotherapy in the future.^41^

In summary, this study highlights a novel engineering strategy to improve γδ T-cell cytotoxicity in the locally inhibitory tumor microenvironment. The results described herein provide a proof-of-concept that PD-1 antibody secretion in γδ T cells enhances checkpoint disruption-associated killing capacity (Fig. 5e). Currently, good manufacturing practice (GMP)-compliant production of Lv-PD1-γδ T cells has been established, and we are preparing for an investigator-initiated trial (IIT) in patients with ovarian cancer in the clinic. In the appropriate clinical setting for solid tumors, adoptive transfer of Lv-PD1-γδ T cells has the potential to surpass natural γδ T cells as a feasible and universal therapy.

## Materials and methods

### Ethics approval

This study collected serial blood samples from human subjects. All experiments were approved by the Chinese Academy of Medical Sciences (CAMS) and the School of Basic Medicine ethical committees (2019010). All patients signed informed consent before they were enrolled in the study. All animal procedures were authorized by the Institutional Animal Care and Use Committee of CAMS. The experiments were performed in accordance with the Animal Research: Reporting In Vivo Experiments guidelines.

### Cell lines and culture

The human ovarian adenocarcinoma cell line OVCAR8, human liver carcinoma cell line HepG2, squamous non-small cell lung cancer cell line NCI-H520, human stomach (gastric) cancer cell line BGC-803, adenocarcinoma human alveolar basal epithelial cell line A549, and adenocarcinoma human breast cancer cell line MDA-MB-231 were all purchased from the Cell Center at the Chinese Academy of Medical Sciences. OVCAR8-GFP-Luc cells were generated by using the plasmid pCDH-EF1-Luc2-P2A-copGFP (Plasmid #72485, Addgene). Using a MofloXDP high-speed flow sorter, stably transfected OVCAR8 cell lines were sorted.

Tumor cells were maintained in RPMI-1640 medium (Catalog No. 30-2001, ATCC). Lentivirus-producing HEK-293T cells were maintained in Dulbecco’s modified Eagle’s medium (DMEM) (ATCC 30-2002). Human γδ T cells were maintained in RMPI-1640 medium with HEPES (N-2-hydroxyethylpiperazine-N-2-ethane sulfonic acid), sodium pyruvate, nonessential amino acids, and 2-mercaptoethanol (Invitrogen). All media were supplemented with 10% heat-inactivated fetal bovine serum (FBS), 2 mM l-glutamine, 100 IU/mL penicillin, and 100 µg/mL streptomycin (Invitrogen).

### Generation of lentiviral constructs

Lentiviral transduction of mammalian cells for high-level protein production was performed as previously described.^42^ The functional variable region of the original sequence of the PD-1 antibody constructed in this study was derived from the humanized monoclonal antibody pembrolizumab (Keytruda^®^). Its heavy chain and light chain were stored in the eukaryotic expression plasmids MH-AbVec2.0-Anti-PD-1-IGHG and ML-AbVec1.1-Anti-PD-1-IGKC, respectively.^43^ The PCR primers used to overlap and amplify the H-chain-little linker-P2A-L chain are as follows. The *EcoR I* and *Xba I* enzyme digestion sites are marked in bold. Then, the overlap product was cloned and inserted into the lentiviral vector PLVX-PGK-IRES-ZsGreen to generate a full-length humanized PD-1 antibody. The expression of the PD-1 antibody was initiated by the PGK promoter. Then the signal peptide was linked to the variable heavy sequence (H-Chain), serine glycine little linker, P2A element, and variable light chain (L-Chain) sequence. IRES-guided ZsGreen served as a fluorescent reporter gene. All constructs were verified by sequencing.

**Table:** 

Primers	Sequences (5′–3′)
H-1F	gtgtcgtgaggatctatttccggt**gaattc**caccatggccgtgctgg
H-1OR	tcgccggcctgcttcagcaggctgaagttggtggcgccgctgcccttgcccagagacagggacagg
L-2OF	tcagcctgctgaagcaggccggcgacgtggaggagaaccccggccccatggcccctgtgcagctgctgg
L-2R	gagaggggcgggatccgcggccgc**tctaga**actagtctcgagctaacactctcccctgttg

### The Biolayer interferometry (BLI) assay

The BLI experiments were designed to detect the binding affinity of the supernatant PD-1 antibody utilizing biolayer interferometry as reported previously.^44^ The preloaded AHC (ahIgG) sensor noncovalently binds to the secreted PD-1 antibody. The loading diluent was PBST (pH 7.4) containing 0.02% Tween-20 and 0.1% BSA, as was the equilibration/solidification buffer. The purified PD-1 protein was serially diluted, and a buffer control was set as background for subtraction at the same time. The loading buffer, immobilized sample, and binding sample (purified PD-1 protein) were added to the sample plate (Greiner PN655209). The program settings included three steps of loading or immobilization, association, and dissociation. The times for these steps were 5 min, 10 min, and 20 min, respectively. The affinity constant (KD) (mol/L) reflects the binding capacity.

### In vitro cytotoxicity testing of γδ T cells by quantitative measurement of LDH

The cytotoxic activity of γδ T cells was determined as previously described.^45^ To measure cytotoxicity, the effector cells, that is, Lv-γδ T cells or Lv-PD1-γδ T cells, were cocultured with tumor cells at the indicated effector: target ratios (10: 1) for 4~12 h and subsequently blended with the Promega G1780 lactate dehydrogenase assay reagent. A plate spectrophotometer was used to measure the lysate absorbance. The target cells were HepG2, OVCAR8, NCI-H520, BGC-803, A549 and MDA-MB-231 cell lines. The killing efficiency was calculated as follows: percent cytotoxicity = 100 × experimental LDH release (OD490)/maximum LDH release (OD490).

### ELISA detection of cytotoxic effector molecules

Changes in the expression levels of IFN-γ, TNF-α, granzyme A, granzyme B, and perforin-1 were detected by ELISA as previously described.^46^ Specifically, expanded γδ T cells were cultured until days 7~9, and the purity was more than 90%. Then, 50 μL of standard and test samples (supernatant from co-culture of armored γδ T cells with tumor cells) were added to the appropriate wells. In the same loading order, 50 µL of the antibody cocktail was added to each well. The ELISA plate was sealed and incubated for 40 min at room temperature (RT) with shaking. Each well was washed 3 times with 1× wash buffer with aspiration from the well. Then, 100 μL of TMB substrate was added to each well and incubated for 5 min at RT in the dark with shaking. Next, 100 μL of stop solution was added to each well and incubated for 1 min while shaking to mix. The absorbance value at A450 nm was recorded immediately, and the expression levels of killing-related effector molecules were compared according to the standard curve.

### Western blot analysis

The supernatant from the transduced γδ T cells was collected and filtered. γδ T cells were lysed using protease inhibitors (Invitrogen) in RIPA buffer (pH 7.5 20 mM Tris-HCl, 150 mM NaCl, 0.5 mM Na_2_ EDTA, 1% sodium deoxycholate, 1% NP-40, and 0.1% SDS) before being centrifuged at 15,000 × *g* for 10 min at 4 °C. Supernatants or whole-cell lysates were loaded onto 10% Mini Protein Gels (Bio-Rad) and then transferred to NC membranes (Bio-Rad). Antibody expression was probed with mouse anti-human IgG (H + L) cross-adsorbed secondary antibody, HRP (ZSGB, ZB-2304), which specifically binds to the heavy chains of human IgG and to light chains common to most human immunoglobulins. Antibody detection was achieved with Pierce ECL Western blot substrate (Invitrogen).

### γδ T-cell tumorigenicity in nude mice

The preclinical safety of Lv-PD1-γδ T cells was tested via an in vivo tumorigenicity assay in nude mice. We assessed the presence of immortalizing or tumorigenic factors in the transgenic cells. The immunodeficient nude mice were randomly divided into 5 groups, namely, the positive control group (NCI-H520), negative control group (1 × PBS), high-dose γδ T group (1 × 10^9^ cells/mouse), medium-dose Lv-PD1 group (1 × 10^7^ cells/mouse), and high-dose Lv-PD1 group (1 × 10^9^ cells/mouse). The above types of cells were inoculated subcutaneously into nude mice (*n* = 5). Observation indicators: The body weight and general physiological state (behavioral activities, physical signs) of the mice were regularly recorded. Anatomical and histopathological examinations were carried out 12 weeks after injection to observe tumor formation at the transplantation site. We prepared another five groups of nude mice to record the survival time within 120 days to support the safety data (*n* = 5).

### In vivo imaging of DiR-labeled γδ T cells

We selected six-week-old adult NOD/SCID/γ null (NSG) mice. They were kept in the Experimental Animal Center of Peking Union Medical College under specific pathogen-free (SPF) conditions. The experimental mice were female, with an initial weight of 20 ~ 22 g. The lipophilic, near-infrared fluorescent cyanine dye XenoLight DiR [DiIC18(7)1,1’-dioctadecyltetramethyl indotricarbocyanine iodide] (710 ex/760 em) was used in this experiment. DiR dye-labeled γδ T cells and Lv-PD1-γδ T cells (1 × 10^7^ cells per mouse) were injected into the caudal vein. The XenoLight DiR^+^ γδ T-cell signal in live animals was periodically tracked by an IVIS Imager in a noninvasive manner. The IVIS Lumina Series III system was used at designated time points after injection (0 h, 2 h, 4 h, 12 h, 1 d, 2 d, 3 d, 4 d, 5 d, 6 d, 7 d, 8 d, 9 d, 10 d, 14 d, 23 d, and 29 d). Another group of mice was used to detect the organ distribution of γδ T cells (*n* = 3). Images of the major organs of each mouse were collected periodically (4 h, 7 d, 29 d). Tissues were formalin-fixed and embedded in paraffin blocks after ex vivo imaging.

### Establishment of an ovarian tumor-bearing mouse model and cellular adoptive immunotherapy

Four-week-old adult NSG mice were housed under specific pathogen-free conditions. The experimental mice were female, with an initial weight of 18~20 g. All procedures were carried out under secure conditions and in strict accordance with the biosecurity regulations approved and supervised by the Animal Care and Use Committee of Peking Union Medical College. OVCAR8-GFP-Luc cells were trypsinized and finally resuspended in 1 × PBS, with the concentration adjusted to 8 × 10^6^ cells/mL. Each NSG mouse received a subcutaneous injection of fifty microliters of the suspension on the back. The mice were randomly divided into five groups (*n* = 10) and received different adoptive immunotherapies. During this process, all mice were monitored for survival, body weight, and tumor volume.

Tumor volume (mm^3^) = *a* × *b*^2^/2, where *a* is the long diameter of the tumor (mm) and *b* is the short diameter of the tumor (mm). The above-mentioned xenogeneic solid tumor mouse models were treated with intratumoral injections (antibody or cell suspension) once every 4 days for a total of seven doses. At the same time, the mice were given an intraperitoneal injection of IL-2 (5000 U/mouse). The specific groups and treatment plans are as follows:

**Table:** 

Groups	Dose/mouse/50 μL
Blank	1 × PBS
γδ T	1 × 10^7^
Lv-γδ T	1 × 10^7^
Lv-PD1-γδ T	1 × 10^7^
γδ T+αPD-1 Ab	1 × 10^7^ + 130 μg

To measure the levels of the anti-PD1 antibodies, ELISA was performed to quantify the levels of human IgG in the serum and tumor tissues of treated mice (Abcam, 195215). The tumors were mechanically dissociated and digested into single cells with collagenase-based buffer before performing flow cytometry to identify specific subsets of tumor-infiltrated γδ T cells with anti-Vδ2^+^ antibodies (anti-human TCR Vδ2 antibody, BioLegend).

Survival curves were analyzed using the Mantel–Cox (log-rank) test.

### Bioluminescence imaging

Imaging was performed with the IVIS Spectrum in vivo imaging system. Anesthesia was provided to the mice as inhaled isoflurane in a gas chamber. Experimental mice were intraperitoneally injected with the substrate D-luciferin (Perkin Elmer, 150 mg/kg). Image acquisition was performed on a 25 cm field of view at the medium binning level with 15-minute exposure times. Analysis was performed using Living Image software (PerkinElmer Biosciences).

### Immunohistochemistry (IHC) and IF

First, tumor tissues from mice were dissected and fixed in prechilled 4% paraformaldehyde for 24 h. Gradient dehydration was performed using the principle of sucrose osmosis until the tissue settled to the bottom. Bubbles were avoided when performing OCT embedding (SAKURA 4583). The embedded tissues were slowly placed into liquid nitrogen with gentle movements to prevent tissue cracks. The samples were stored in a −80 °C freezer, and frozen sections with a thickness of 8~10 μm were prepared with a Leica Rotary Microtome (CM1950). The labeled slides were placed in a constant temperature incubator at 65 °C for 2 h and dried at room temperature. Then, they followed permeabilization with 0.1% Triton X-100 in 1× PBS and blocking with 10% normal goat serum (Boster AR0009). We used TCR gamma/delta monoclonal antibody (Invitrogen 5A6. E9), anti-CEA antibody [EPCEAR7] (Abcam ab133633), and anti-NSE antibody [EPR3377] (Abcam ab79757) as primary antibodies. After that, we diluted the goat serum appropriately and incubated the sections for an hour at room temperature in a humid, dark environment.

The slices were next treated for 45 min with biotinylated secondary antibodies (Vector Laboratories MP-7402) for tissue IHC before being developed for three washes with Immpress HRP reagents. Prior to cover-slipping, the nuclei were lastly stained with Mayer’s hematoxylin for 5 min. Target proteins were detected by tissue IF using DyLight^®^ 488-anti-mouse IgG and Alexa Fluor 555-anti-human IgG (H + L). ProLong Gold Antifade Mountant with DAPI (Invitrogen F6057) was used to coat the sections.

Leica DM6 B microscope scanning was carried out on the IHC sections. IF images were taken with a Zeiss LSM 780 confocal microscope or scanned with a Leica DMi8. Zeiss ZEN Service Blue software was used to analyze IF pictures. For IF/IHC statistics, we applied ImageJ software to measure the average density (average optical density, AOD) or the number of positive cells visible per unit area covering the area.

### Statistical analyses

All representative experiments were repeated at least three times. All statistical analyses were performed using an unpaired, two-tailed *t test* (a *p* value < 0.05 was considered significant). All calculations were performed with Prism 8.0 (GraphPad) software. Data are presented in the results and figures as the mean ± SEM.

### Supplementary information

Supplementary information - Clean version

## Data Availability

The data used in the current study are available from the corresponding authors upon reasonable request.
