# Supplementary material for: Anti-PD-1 antibody armored γδ T cells enhance anti-tumor efficacy in ovarian cancer
Source: Signal Transduct Target Ther. 2023 Oct 20;8:399. doi: 10.1038/s41392-023-01646-7 (PMC10587135; doi:10.1038/s41392-023-01646-7)
Supplement: Supplementary file 1 — Supplementary information - Clean version [file 41392_2023_1646_MOESM1_ESM.docx]

Supplementary Materials for

# Anti-PD-1 antibody armored γδ T cells augmented anti-tumor efficacy in ovarian cancer

Yue Wang^1^, Jingyi Han^1,2^, Dongdong Wang^1^, Menghua Cai^1^, Yi Xu^1^, Yu Hu^1^, Hui Chen^1,3,4^, Wei He^1,^ *, Jianmin Zhang^1,3,4,^ *

Correspondence to: Dr. Wei He at E-mail: [heweingd@126.com](mailto:heweingd@126.com); Dr. Jianmin Zhang at E-mail: [jzhang42@163.com](mailto:jzhang42@163.com)

**This PDF file includes:**

Materials and Methods

Figures S1 to S4

**Materials and Methods**

## Isolation of human peripheral blood mononuclear cells (PBMCs) and modification of γδ T cells

A disposable vacuum blood collection needle was prepared to aseptically collect peripheral venous blood from the donor’s elbow. Fresh human PBMCs were separated by density gradient centrifugation on Ficoll-Hypaque (tbdscience TBDTM-0050). The cell suspension concentration was adjusted to 4 × 10^6^ cells per well in 24-well plates coated with an anti-TCR pan-γδ antibody (Beckman Coulter IM1349, 2 ng/mL) to expand pure γδ T cells. The cells were cultured in typical 5% CO_2_ incubators run at 37°C. Four days later, the cells were transferred to uncoated wells and passaged according to growth density. The γδ T cells were cultured *in vitro* for 7~9 days before lentiviral infection. To increase the viral infection efficiency of the primary γδ T cells, the culture plates with γδ T cells were centrifuged after the addition with lentiviral supernatant and protamine sulfate (Sigma‒Aldrich P3369, 10 μg/mL) at 1000 × g for 90 minutes (MOI = 12). Then the cells were placed in a 37°C, 5% CO_2_ incubator and replaced with complete medium after 12 hours. The cytological verification was performed 72~96 hours after lentiviral infection. These above cells modified by the PD-1 antibody lentivirus and the nonsense vector lentivirus were named Lv-PD1-γδ T cells and Lv-γδ T cells, respectively.

## Lentiviral packaging and purification

We used the target PLVX-PGK-PD-1 Ab-IRES-ZsGreen or vector PLVX-PGK-IRES-ZsGreen with pMD2G (VSV-G envelope-expressing plasmid) and psPAX2 (HIV-1 gag pol-expressing plasmid) to obtain high-quality DNA for packaging lentiviruses and ensure the removal of endotoxin that is required for successful transfection into HEK-293T cells. HEK-293T cells were removed from liquid nitrogen and passaged less than ten times for the best transfection potential. 293T cells were plated to achieve 80~90% confluency on the day of transfection. We used jetPRIME^®^ (Polyplus, PT-114115) for cotransfection of multiple plasmids of lentivirus production.

Then, the cells were incubated at 37°C in 5% CO_2_ for 72 hours and subjected to virus purification and titration. The lentivirus was concentrated and purified by ultracentrifugation methods. The culture supernatants of HEK-293 T cell were centrifuged at 650 × g for 5 minutes at 4°C to remove cell debris and filtered using a 0.45 μm filter. Then the virus fluids were transferred into a pre-cooled sterile Amicon Ultra-15 100K centrifuge filter and centrifuged at 5000 × g for 30 minutes before being stored in a -80°C freezer. The lentiviral titers were determined by real-time quantitative PCR. Total RNA from cell samples with different amounts of lentivirus was extracted and reverse transcribed into cDNA. Absolute quantification was then performed on the *Ct* values of the detection targets, WPRE and ACTB. Typically, a difference in *Ct* values of 2-fold or more is considered significant. Another lentiviral titer was determined by serial dilution method. Lentivirus titer = number of fluorescent cells/volumes of virus stock solution.

## Reconstitution of humanized mice and flow cytometry analysis

Fresh human PBMCs were separated by density gradient centrifugation on Ficoll-Hypaque (tbdscience TBDTM-0050) and suspended in RPMI-1640 medium. Each NSG mouse was intravenously transfused with 2 × 10^7^ of cell suspension via the caudal vein. Peripheral blood from reconstituted PBMC humanized (Hu-PBMC) mice and NSG mice were isolated 24 hours and 1 week after intravenous injection of PBMCs and analyzed by flow cytometry. Cells were incubated with purified anti-human CD45 antibody (Clone QA21A24, Biolegend) at 4°C for 45~60 minutes. The stained cells were washed by 1 × PBS before being analyzed by flow cytometry.

Figure S1


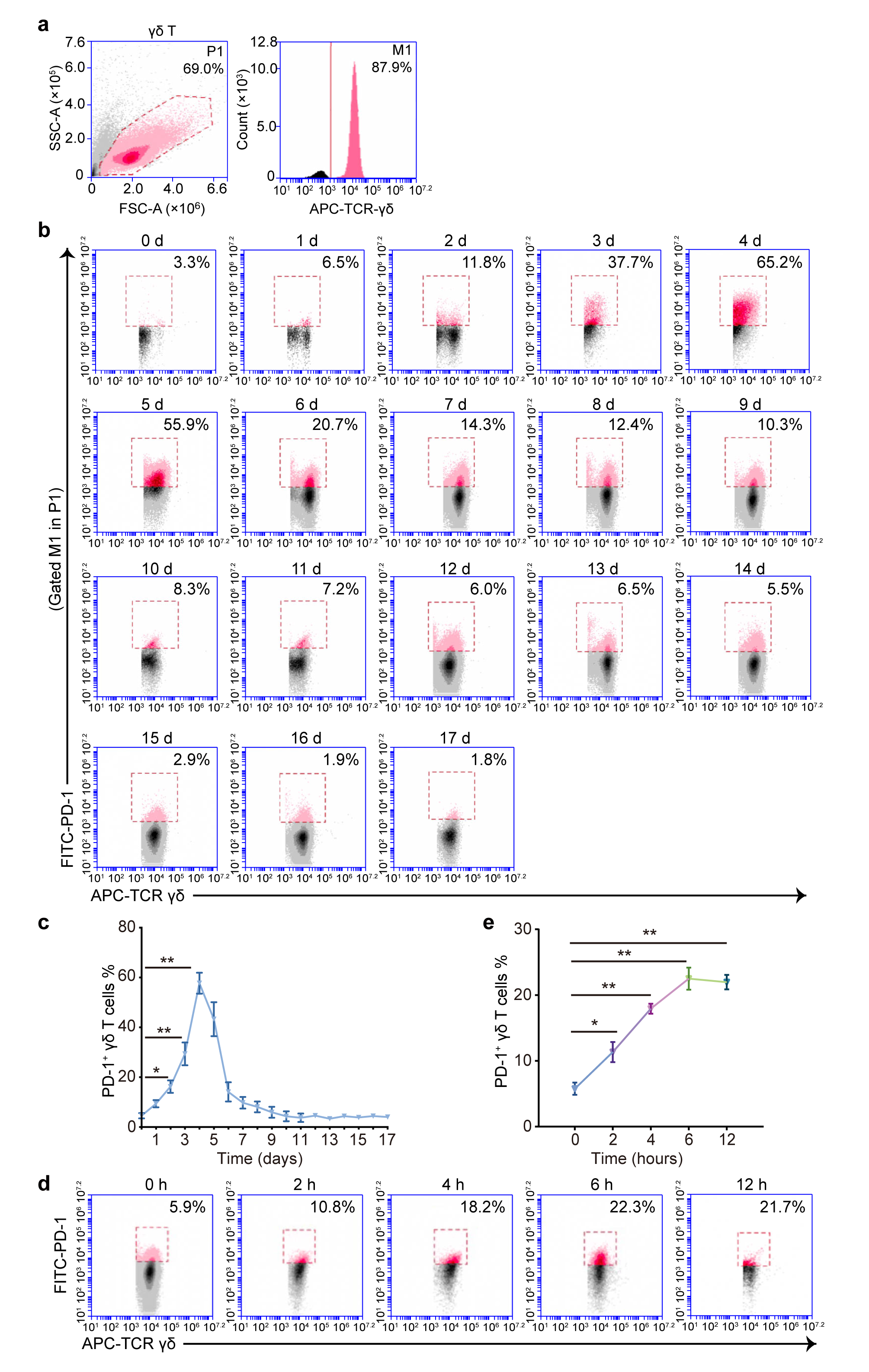


## Figure S1. The levels of PD-1 expression on γδ T cells were upregulated when activated by pan-antibodies or cocultured with tumor cells in vitro

γδ T cells were expanded from freshly isolated human PBMCs in the wells precoated with anti-TCR pan-γδ antibodies for the first 4 days. Then, the cells were transferred to a new plate to amplify without anti-TCR pan-γδ antibody stimulation. **(a)** Flow cytometry was performed to examine the purity of γδ T cells and the levels of PD-1 expression on the γδ T cells. The cells were gated based on the cell size and APC-TCR fluorescence for γδ T cells and FITC fluorescence for PD-1 expression. **(b, c)** Representative flow cytometry plot **(b)** and statistical analysis **(c)** of the frequencies of PD-1-expressing γδ T cells (*n* = 3). **(d, e)** Representative flow cytometry plot **(d)** and quantification **(e)** demonstrating increased PD-1 detection on γδ T cells following coculture with HepG2 tumor cells (*n* = 3). The detection time points were 0 hr, 2 hr, 4 hr, 6 hr, and 12 hr. Data are represented as the mean ± SEM. *, *p* < 0.05; **, *p* < 0.01.

Figure S2


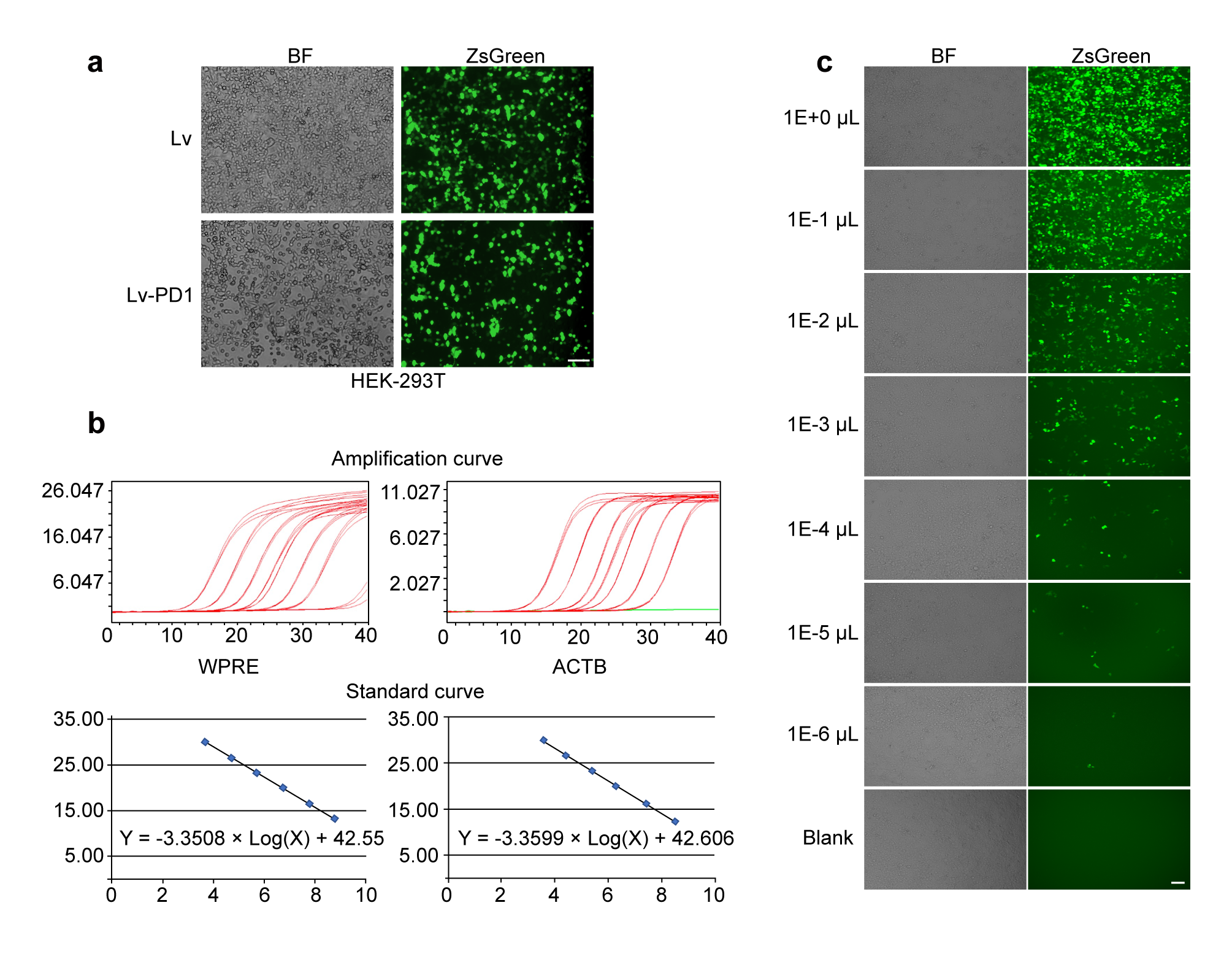


## Figure S2. Preparation of lentivirus expressing PD-1 antibodies

**(a)** Representative images show fluorescence detection after lentivirus packaging of HEK-293T cells with lentivirus carrying the vector (Lv) or the target PD-1 antibody (Lv-PD1), showing the BF control (white light) and ZsGreen (green fluorescence). Scale bars, 100 μm. **(b)** The lentiviral titers were determined by real-time quantitative PCR. Total RNA from cell samples with different amounts of lentivirus was extracted and reverse transcribed into cDNA. Absolute quantification was then performed on the *Ct* values of the two detection targets, WPRE [Y = -3.3508 × Log (X) + 42.55] and ACTB [Y = -3.3599 × Log (X) + 42.606]. Typically, a difference in *Ct* values of 2-fold or more is considered significant. **(c)** Lentiviral titers were determined using a serial dilution method. Scale bars, 100 μm. Lentivirus titer = number of fluorescent cells/volumes of virus stock solution.

Figure S3

^
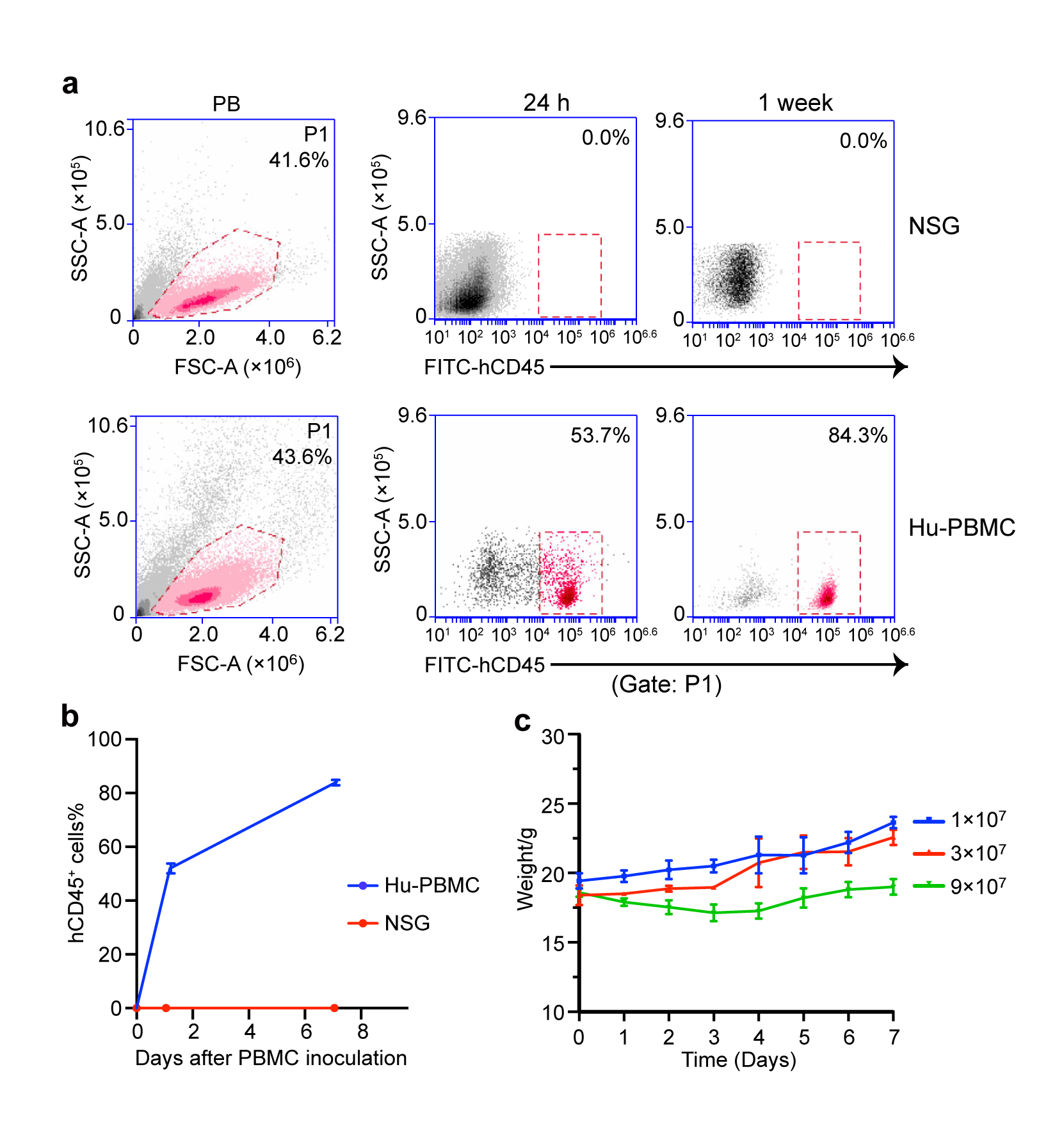
^

**Figure S3. The safety of Lv-PD-1 γδ T cells in humanized NSG mice**

**(a)** Flow cytometry to analyze the peripheral blood (PB) of reconstituted Hu-PBMC mice and NSG mice 24 hours and 1 week after intravenous injection of PBMCs. The cells were gated based on the cell size and FITC fluorescence for human CD45 (*n* = 3). **(b)** Quantification of the percentage of human CD45^+^ cells shown in the panel **a** (*n* = 3). Data are represented as the mean ± SEM. **(c)** The weight change curves of Hu-PBMC mice injected intravenously with different doses of Lv-PD1 γδ T cells.

Figure S4


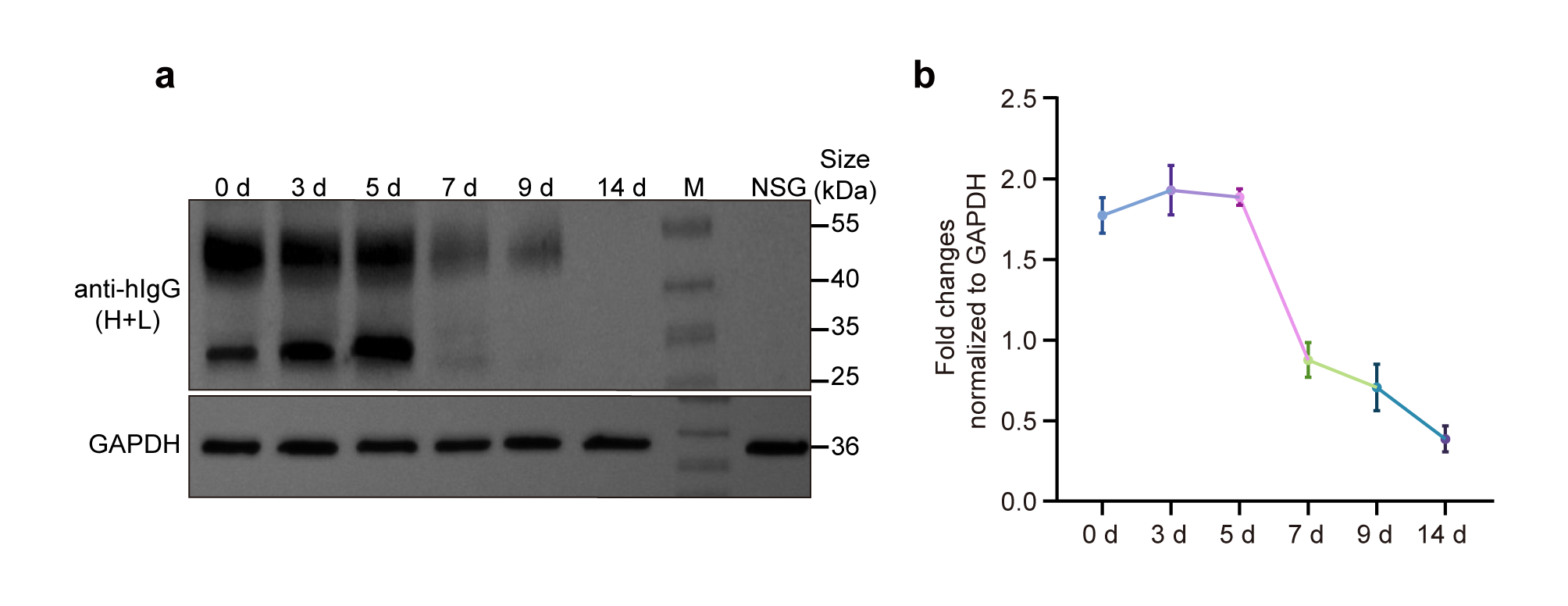


## Figure S4. Lv-PD1-γδ T cells could produce anti-PD-1 antibodies for up to 9 days in vivo

For analysis of PD-1 Ab levels in tumors, OVCAR8 cells were subcutaneously (s.c.) inoculated on the right side of the back of NOD/SCID/γ null mice (*n* = 7). **(a)** Each tumor-bearing mouse was ultimately subjected to time-point dissection at day 0, day 3, day 5, day 7, day 9, day 14. Western blot analysis was performed to examine the *in vivo* attenuation of hIgG and the metabolism of intratumorally injected Lv-PD1-γδ T cells (1 × 10^7^/mouse). Each mouse was sacrificed to remove the tumor at the same time-point. The number of mice killed under each condition was equal. GAPDH served as a protein loading control. **(b)** Quantification of the levels of hIgG expression of the Western blot shown in the panel **a.**
